# Colorectal cancer in a region of western of Algeria: results of 581 cases in 5 years

**DOI:** 10.4314/ahs.v23i2.39

**Published:** 2023-06

**Authors:** Bouchra Dahmani, Lamia Boublenza, Naffissa Chabni, Dalale Behar, Hafida Hassaine, Nabila Masdoua, Amira Nahet, Kaouel Meguenni, Ilyes Zetla

**Affiliations:** 1 Laboratory of Microbiology Applied to the Food Industry, to Biomedical and to the Environment (LAMAABE), Department of Biology, University of Abou Bekr Belkaid, Tlemcen, Algeria; 2 Department of Epidemiology, Dr Tidjani Damerdji University Hospital, Tlemcen, Algeria; 3 Cancer Lab N° 30 Laboratory, University of Abou Bekr Belkaid, Tlemcen, Algeria

**Keywords:** Colorectal cancer, epidemiology, Algeria, Tlemcen province

## Abstract

**Aims:**

The objective of this work is to evaluate the epidemiological profile of colorectal cancers, histologically proven over a 5-year period (2012-2016) in the Tlemcen region.

**Methods:**

A retrospective study of 581 cases of colorectal cancer collected at the epidemiology department of the University Hospital Center (UHC) of Tlemcen between January 2012 and December 2016 was performed. Epidemiological data were processed using SPSS version 25 and Microsoft Excel 2010.

**Results:**

The epidemiological profile has shown that colorectal cancer in our region ranks 3^rd^ in both sexes. There were 322 men (55.4%) affected compared to 259 women (44.6%) with a sex ratio of 1.2. A predominance of males is noted in 50-60 age group, while for the female sex, the dominance is between 60-70 years old. The mean age of CRC occurrence was 60±13 years, with an extremity ranging from16 to 90. A significantly higher rate was recorded for rectal cancer (43.7%) followed by sigmoid colon (5.7%). Variable rates were recorded during the 5 years with a peak in 2014 (27.9%).

**Conclusion:**

Regular analysis of these data, if supplemented with additional data on diagnostic modalities like circular RNA diagnostic, will contribute to the assessment of the impact over time of public policies on nationally organized CRC screening.

## Introduction

Colorectal cancer (CRC) is a major public health concern around the world. It is the third most common malignant neoplasm in the world and the second most common cause of cancer death, with an estimated 935,000 deaths in 2020; of which 54.2% occurred in Asia, 26.2% in Europe, and 4.6% in Africa^1^.

The term colorectal cancer refers to a slow-growing cancer that begins as a tumor or tissue growth on the inner wall of the rectum or the colon. If this abnormal growth, called a polyp, ends up becoming cancerous, it can form a tumor on the wall rectum or colon and subsequently develop into blood or lymphatic vessels, increasing the risk of metastasis to other anatomical sites 2-3.

The incidence of CRC varies significantly from country to country depending on their economic development but the standard myth of colorectal cancer being a disease limited to western countries need to be dispelled 4.

In Algeria, after an annual meeting of the National Cancer Registries Network held in Algiers in 2020, Pr Bouzid announces that colorectal cancer in women over 40 is the most common after breast cancer, and the first cancer common in men, well before that of lung^5^-^6^.

The objective of this work consists of assess the epidemiological profile of colorectal cancers, histologically proven over a of 5 years period (2012-2016) in Tlemcen region.

## Methods

### Study design

A retrospective study of 581 cases of colorectal cancer collected at the Epidemiology Department of the University Hospital Center (UHC) of Tlemcen between January 2012 and December 2016. more than 70% of the cancers are diagnosed by histopathology Demographic variables studied were: age at the time of diagnosis, gender. The clinical characteristics included are; clinical presentation, tumor site, stage and type of the tumor.

### Inclusion and exclusion criteria

Only data from the cancer registry of the Epidemiology Department of the University Hospital Center Dr. Tidjani Demerdji in Tlemcen have been included, therefore All histologically confirmed colon and rectal cancers were included.

While all data from other registries have been excluded, thus All duplicate cases.

### Data analysis

The data collected were organized and analysed with SPSS version 25 software and Microsoft Excel 2010.

This study was approved by the ethics committee of Dr Tidjani Damerdji University Hospital-Tlemcen (173.FM. UABB.23).

## Results

The evaluation of the epidemiological profile has shown that colorectal cancer in our region is the 3rd most incident cancer after breast and thyroid cancer in women with 10.7 / 100,000 inhabitants and after bladder and prostate cancer in men with 14.3 / 100,000 inhabitants (Table 1).

**Table 1:** The incidence rate of the top 10 cancers by sex from 2012 to 2016

Type of cancer	Incidence (Male)	Incidence (Female)
Breast	-	61.9
Colon, rectum, anus	14.3	10.7
Lymphoma	11.3	8.8
Bladder	17.9	2.2
Stomach	9.4	6.1
Thyroid	2.6	12.8
Lung, trachea, bronchus	12.6	2.0
Prostate	14.4	
Brain & nervous system	5.3	5.8
Mouth & pharynx	7.1	2.9

The distribution of colorectal cancer by sex indicates that the male population is the most affected; since out of the 581 patients analysed, 322 men are affected (55.4%) against 259 women (44.6%), with a sex ratio of 1.2.

**Figure 1:**
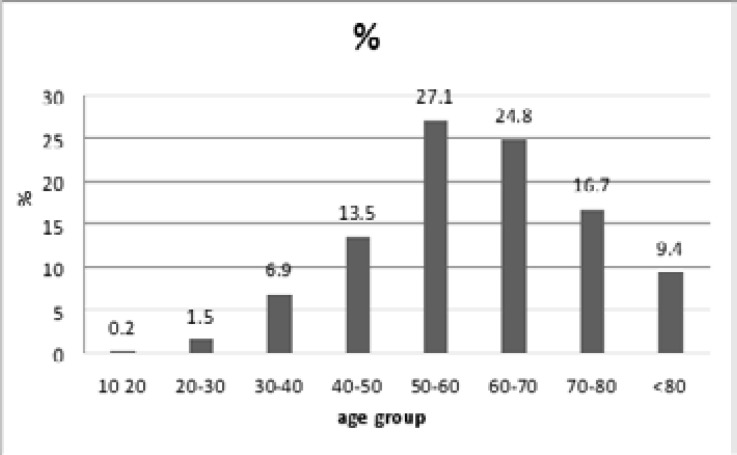
distribution of colorectal cancer by age group percent

Figure 1 shows that the age group most represented in this study is that of 50-60 with 27.1%, followed by that of 60-70 with 24.8%. The mean age of onset of colorectal cancer was 60±13 years, with an extremity ranging from16 to 90.

**Figure 2:**
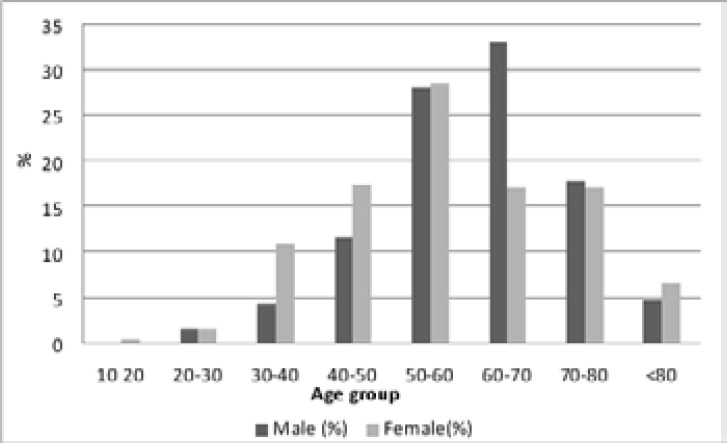
Distribution of colorectal cancer by sex and age groups

When gender is considered, a predominance of males is noted in 50-60 age group, while for the female sex, the dominance is between 60-70 years old (Figure 2).

**Figure 3:**
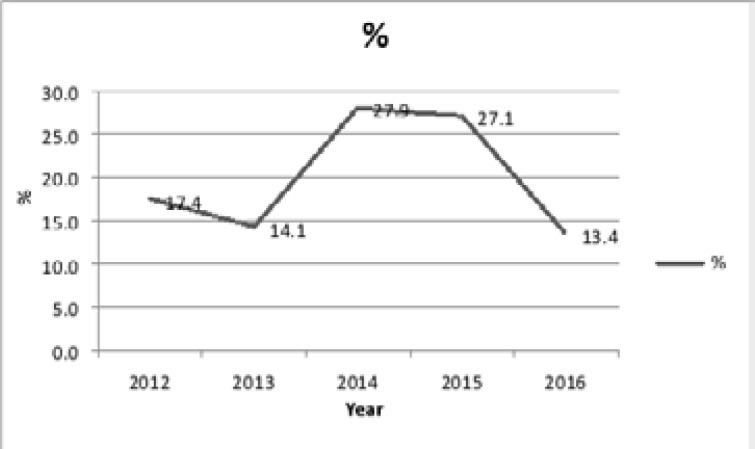
Year wise distribution of colorectal cancer cases

Variable rates were recorded during the 5 years (Figure 3) with a peak in 2014 (27.9%), and an annual average of 116 cases for a total of 581 cases. What was also noticed is the significant decrease in these cancers in 2016 with 13.4%.

**Figure 4:**
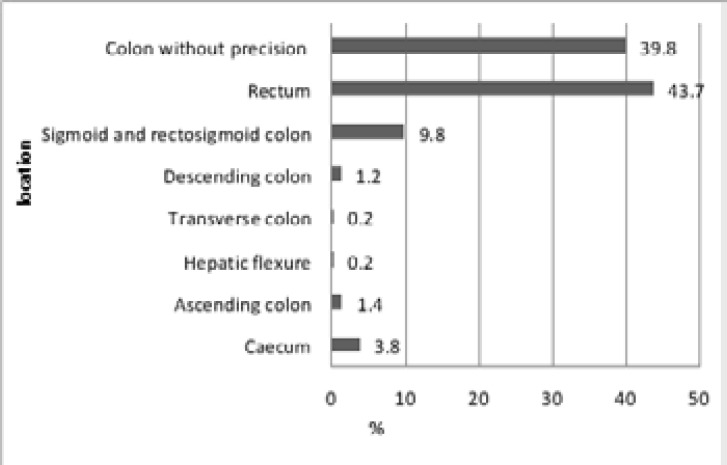
Distribution of colorectal cancer according to location

This statistical analysis carried out on the whole target population studied allowed us to show a significantly higher rate for rectal cancer (43.7%) followed by the sigmoid colon (5.7%), the rectosigmoid junction (4.1%) and the caecum (3.8%) while 39.8% of colon cancers were unspecified (Figure 4).

In this analysis (Table 2), adenocarcinoma appears to be the most predominant type of cancer with 82,79%, followed by carcinoid with 6,37%.

**Table 2:** Distribution of colorectal cancer according to morphology

Morphology	N (%)
Carcinoid	37(6,37)
Adenocarcinoma	481(82,79)
Melanoma	2(0,34)
Sarcoma	1(0,17)
Others	60(10,33)

According to AJCC 8th edition staging, a rate of 26.3% was on stage I, 17.4% of patients presented with stage IV while 38.5% were at an undetermined stage (Table 3).

**Table 3:** Distribution of colorectal cancer by stage of diagnosis

Stage of diagnosis	N (%)
Stage I	153(26,3)
Stage **II**	103(17,8)
Stage **III**	0(0%)
Stage **IV**	101(17,4)
Undetermined	224(38,5)

## Discussion

Colorectal cancer is a real public health problem, both in Algeria and the whole world by its frequency and high mortality. More than a third of new cases of colorectal cancer occur outside of the industrialized countries^9^.

The CRC incidence rate increased by 90% from 1980 to 2016, this development is largely attributable to the constellation of dietary and lifestyle factors such as poor diet characterized by high intakes of red and processed meat and alcoholic drinks and low intakes of fiber-rich foods, low physical activity, and obesity^7^.

The evaluation of the epidemiological profile has shown that colorectal cancer in our region is the 3^rd^ common cancer after breast and thyroid cancer among females (10.7 / 100,000 inhabitants) and after bladder and prostate cancer among males (14.3 / 100,000 inhabitants). The incidence of colorectal cancer in our region remains higher than that of Morocco (2.5 to 3.3/100,000 inhabitants)^8^, Egypt (6.3/100,000 inhabitants and 6.6/100,000 inhabitants for women and men respectively) and India (3.1/100,000 inhabitants and 5.8/100,000 inhabitants for women and men respectively)^9^.

However, it is lower than that of western countries including the United States of America (22.6/100,000 inhabitants among females and 28.8/100,000 inhabitants among males), Germany (21.1/100,000 inhabitants among females and 31/100,000 inhabitants among males), Canada (28/100,000 inhabitants among females and 35.2/100,000 inhabitants among males) and France (24/100,000 inhabitants among females and 36.9/100,000 inhabitants among males )^9^.

The GLOBOCAN age-standardised estimated incidence rate shows Australia and New Zealand as having the highest rates of CRC in the world with 44/100,000 inhabitants among females and 56/100,000 inhabitants among males and 37.5/100,000 inhabitants among females and 44.1/100,000 inhabitants among males respectively^10^-^11^.

In our study, 581 cases of CRC were found over a period of 5 years. This result is higher than those found in the African literature, a study carried out in Togo declared 57 cases of colorectal cancer during 10 years12, another study from Gabon declared 51 cases of this cancer over 10 years13. The male predominance observed in our study (55.4%) with a sex ratio of 1.2 was consistent with the results of several authors, thus confirming the data in the literatur^12^-^14^.

Age is one of the factors involved in the appearance of these cancers, and in this work the average age of cancer onset was 60.05 years which is in agreement with a study carried out in Morocco in 2015^15^, another study carried out in Asia whose average age was 61 years which is similar to our result^16^. On the other hand, studies carried out in sub-Saharan Africa show that colorectal cancers affect particularly the younger population whose average age is 48 years and 46.7 years recorded in Gabon and Togo respectively^12^-^13^.

Conversely the average age of our population is even younger than the corresponding age in the United States (69 for men and 73 for women)^17^, and in France which declares an average age which is in the seventh decade. The number of new cases of colorectal cancer estimated was 31.2% occurring in patients aged from 65 to 74 years and 42.5% in patients aged 75 and over^18^. The highest rate was observed in the interval of 50-60 years with 27.1%, which confirms a study that was carried out on a population of western Algeria^19^, another Moroccan study states the same most affected age group^12^.

In addition; in our series, the cancer site in order of frequency was the rectum (43.7%), sigmoid colon (5.7%), rectosigmoid junction (4.1%) and cecum (3.8%) which is in agreement with a study carried out in Iraq in 2019^18^. Another study in Iran indicated that the rectum (31.1%), the sigmoid colon (20.4%) and the ascending colon (18.6% ) were the most common tumor sites^21^, whilst our series do not agree with the literature done in Algeria whose Malignant involvement of the left colon shows a predominance with 61.8%^17^, and even with another study done in Jordan in 2018 which marks a dominance of Malignant involvement of the left colon with 77.12%^22^.while 39.8% are colon cancers without precision, which represents a limit of the study.

Adenocarcinomas are the histological variety that dominates colorectal cancers in our series with 82,79% of cases. This Figure is compatible with those reported in other studies carried out in Togo (91.2%), Gabon 98%, Morocco (95%), Egypt (76.2%), Saudi Arabia (81%), Iraq (84.0%) and Iran with 96.4%^8^,^9^,^13^,^23^,^24^,^25^.

The descriptive analysis of the abnormalities showed a 26.3% rate of cancers that were at a stage I, 17.8% at a stage II and 17.4% at a stage IV which is consistent with a study carried out by the cancer registries of the Francium network in France^26^.

## Conclusion

The evaluation of the epidemiological profile has shown that colorectal cancer in the region of Tlemcen is the 3rd most incident cancer after breast and thyroid cancer in women and also the most common one after bladder and prostate cancer in men with an average age of 60±13 years and age extremes of 16 to 90 years and a predominance of colon cancer (56.3%) compared to rectal cancer (43.7%).

Regular analysis of these data, if enriched with additional diagnostic methods like circular RNA method which have relatively high diagnostic accuracy in distinguishing CRC patients from normal controls25, would help to assess the impact over time of public screening policies organized at national level for colorectal cancer and they will provide useful information for the various work of clinicians and researchers.
