# Erratum to: colorectal cancer in a region of western of Algeria: results of 581 cases in 5 years

**DOI:** 10.4314/ahs.v23i3.89

**Published:** 2023-09

**Authors:** Bouchra Dahmani, Lamia Boublenza, Naffissa Chabni, Dalale Behar, Hafida Hassaine, Nabila Masdoua, Amira Nahet, Kaouel Meguenni, Ilyes Zatla

**Affiliations:** 1 Laboratory of Microbiology Applied to the Food Industry, to Biomedical and to the Environment (LAMAABE), Department of Biology, University of Abou Bekr Belkaid, Tlemcen, Algeria; 2 Department of Epidemiology, Dr Tidjani Damerdji University Hospital, Tlemcen, Algeria; 3 Cancer Lab N° 30 Laboratory, University of Abou Bekr Belkaid, Tlemcen, Algeria

## Erratum

After publication of the original article (reference number: WKR0-2022-01-0085.R4), it came to the author's attention that there was a typing error in the one of the author names.^1^

 

The published name

Ilyes Zetla.

 

The correct name

Ilyes Zatla.

 

Publication issue

Volume 23, Issue 2
